# CXCL16 knockout inhibit asthma airway inflammation by suppressing H2-DM molecular mediated antigen presentation

**DOI:** 10.1038/s41420-025-02371-6

**Published:** 2025-03-06

**Authors:** Ting-ting Liu, Zhi Zhang, Jing Deng, Chang-yu Shi, Shuai Zheng, Li-xin Jia, Jie Du, Chunmei Piao

**Affiliations:** 1 Beijing Anzhen Hospital, Capital Medical University, Beijing, China; 2 Beijing Institute of Heart, Lung and Blood Vessel Diseases, 100029 Beijing, China; 3 The Key Laboratory of Remodeling Cardiovascular Diseases, Ministry of Education, Beijing, China; 4 Collaborative Innovation Center for Cardiovascular Disorders, Yanji, China; 5 School of Basic Medical Sciences, Yanbian University, 133000 Yanji, China

**Keywords:** Cell biology, Antigen-presenting cells

## Abstract

The inflammatory microenvironment influences dendritic cell-mediated antigen presentation to regulate asthma Th2 inflammation. The scavenger receptor is expressed on DCs and regulates antigen presentation and T priming. However, whether the transmembrane scavenger receptor (SR-PSOX/CXCL16) regulates the phenotype and antigen presentation function of DCs remains unclear. We found that CXCL16 is mainly expressed on DCs in the lung tissues of asthma patients and asthma mice. CXCL16 knockout led to the suppression of airway inflammation, mucus overproduction, and airway hyperresponsiveness in *Aspergillus*-induced asthma. In addition, the adoptive transfer of *Aspergillus*-pulsed DCs shows the CXCL16^+^ DCs exerted a promoting role in airway inflammation, the CXCL16^−^ DCs inhibit airway inflammation. Additionally, RNA sequencing and flow cytometry data revealed that CXCL16 knockout inhibits airway inflammation by suppressing the antigen processing and presentation function of DCs, which was mediated by MHC II chaperone H2-DM. Furthermore, we found CXCL16 knockout suppressed dendritic cells differentiated forward to cDC2b subtype which is mainly charged with antigen presentation to T cell. In conclusion, we found that CXCL16 downregulated the capacity of DC antigen processing and presentation to suppress airway inflammation by reducing H2-DM expression which mediated DC differentiation. The study suggested that inhibition of CXCL16 can be a potential therapy for asthma.

## Introduction

Asthma is a chronic airway inflammatory disease characterized by the infiltration of inflammatory cells, excessive secretion of mucus, airway hyperresponsiveness, and airway contraction. A meta-analysis indicates that the prevalence of *Aspergillus fumigatus* sensitization in children with asthma is 16.1%, which is much higher than that of allergic bronchopulmonary aspergillosis [1]. There is a significant correlation between the prevalence of indoor asthma in children and adolescents and the level of *A. fumigatus* [2]. Compared with exposure to lower concentrations of fungi, exposure to higher concentrations of *Aspergillus* and other fungi increased the aggravation of asthma symptoms to 36–48% [3]. In the asthma animal model induced by *A. fumigatus*, the production of effector cytokines can activate Th cells, produce different types of inflammatory reactions (such as Th1, Th2, Th9, and Th17), or exhibit mixed Th2/Th17 inflammatory reactions [4, 5], which are related to the different proteinaceous antigens used in *Aspergillus* [6–8] and dendritic cells (DCs) subset antigen presentation [9]. Therefore, in asthma induced by *A. fumigatus*, studying the process of DC priming to T cells will play an important role in preventing and treating the inflammatory response caused by asthma.

Previous studies have found that, as antigen-presenting cells, DCs have a strong ability to perceive tissue damage, capture antigens, and present processed antigens to T lymphocytes. At the initial stage of an inflammatory response, DCs capture antigens through endocytosis or phagocytosis, activate DCs, differentiate into different cell subpopulations, present peptides on MHC II protein complexes, induce naïve T cells to activate and differentiate into Th2 cells, and then release the Th2 cytokines IL-4 and IL-13, which trigger a series of immune system activation events [10]. The process of dendritic cell activation is influenced by the inflammatory microenvironment, thereby altering the main phenotype and function, particularly affecting antigen capture, processing, and MHC II complex transport [11]. Previous studies have found that the scavenger receptor (SR) expressed on DCs regulates antigen presentation and T priming, such as CD36 (class B scavenger receptors) and lectin-like oxidized low-density lipoprotein receptor-1 (LOX-1) (class E scavenger receptor), thereby regulating the inflammatory response [12, 13]. These processes are related to autophagy and mitochondrial metabolic reprogramming. However, it is unclear whether another transmembrane scavenger receptor (SR-PSOX/CXCL16) regulates the phenotype and antigen presentation function of DC.

The scavenger receptor CXCL16 is a type I transmembrane protein. As a scavenger receptor, CXCL16 can bind and internalize modified forms of oxidized low-density lipoprotein, which is crucial for removing apoptosis cells and the uptake of various pathogens [14]. Its extracellular region has a chemokine domain, and under proteolytic cleavage of metalloproteinase ADAM10, it generates the soluble-(s) chemokine sCXCL16 to exert chemokine function. In asthma airway inflammation, dendritic cells express a high level of CXCL16 under the condition of DC maturation and proinflammatory stimulation [15]. CXCL16 expressed in mature DCs recruits lymphocytes through the chemokine receptor CXCR6 and communicates with lymphocytes [16]. In addition, dendritic cells release CXCL16 induced by Toll-like receptor 7 activating neutrophils and secreting cytokine IL-8 [17]. These studies provided a certain understanding of CXCL16 expressed on DCs, but the exact function of CXCL16 in asthma airway inflammation and whether CXCL16 expressed on dendritic cells can regulate airway inflammation in *Aspergillus*-induced asthma models are still unclear.

In this study, we investigated the pathophysiological role of CXCL16 expressed on dendritic cells in *Aspergillus*-induced airway inflammation to elucidate the function of CXCL16 in DC priming T-cell inflammation. This is the first study to demonstrate that CXCL16-knockout mice had reduced airway inflammation through dysfunction of DCs priming T cells, independent of its role in chemotaxis.

## Results

### CXCL16 expressed in DCs and is increased in asthma inflammation

**Fig. 1:**
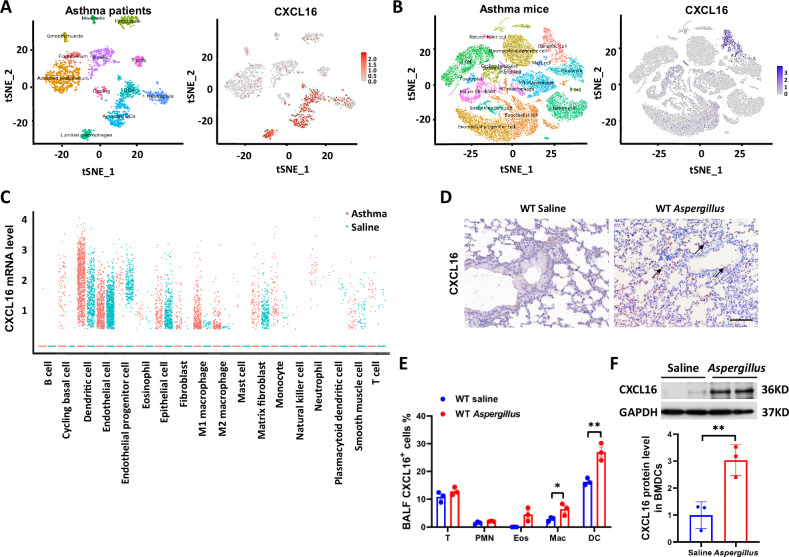
Dendritic cell CXCL16 was increased in *Aspergillus* challenge asthma. **A** Single-cell RNA-sequencing analysis showed the distribution of CXCL16 expression in the lung tissue cell subgroups of asthma patients: the left figure is a tSNE plot of the single-cell RNA sequence dataset annotated by cell type in the Lung Cell Atlas; the right figure is a tSNE diagram characterized by the distribution of CXCL16-positive cells in the airway. **B** The scRNA-seq analysis of OVA-induced asthma mice: the left figure is a cell subset of asthma lung of mice; the right figure shows the distribution of CXCL16-positive cells in OVA-induced asthma lung. **C** The mRNA expression of CXCL16^+^ cells in each cluster from the scRNA-seq of asthma mice. **D** Lung sections were stained with immunohistochemical staining of CXCL16 to show CXCL16 positive cell infiltration (×200 magnification and scale bar = 100 μm). **E** Percentage of CXCL16^+^ cell expression was assessed using flow cytometry on inflammatory cells in bronchoalveolar lavage. PMN neutrophils, Eos eosinophils, DC dendritic cells, Mac macrophages (*n* = 3). **F** The protein expression level of CXCL16 in BMDCs detected by western blot (*n* = 3). Data shown are mean ± SEMs. *P < 0.05, **P < 0.01 wild-type mice challenged with *Aspergillus* versus treated with saline.

To explore the role of CXCL16 in the pathogenesis of asthma, we first clarified the localization of CXCL16 and assessed the distribution of CXCL16-positive cells in the lung tissue. Lung Cell Atlas scRNA-seq dataset of asthma patients was used to show the expression of CXCL16^+^ cells in lung tissue. We found that CXCL16 predominated distributed in DCs, activated DCs, and luminal macrophage in asthma patients (Fig. 1A). We obtained similar results from scRNA-seq analysis in mice asthma models (Fig. 1B). Then, we calculated the mRNA expression of CXCL16 in each cell cluster of asthma lung, and we found that the mRNA expression of CXCL16 in DCs and endothelial cells were increased in mice asthma model. (Fig. 1C). We performed immunohistochemical staining of CXCL16 on lung tissue sections of the asthma model group, and the results showed that in addition to being expressed in epithelial cells, CXCL16 was also highly expressed in certain inflammatory cells infiltrating the lungs, as shown in Fig. 1D. According to literature reports, CXCL16 is expressed in macrophages, peripheral lymphocytes, dendritic cells, and epithelial cells [18–20], and CXCL16 was discovered in bronchoalveolar lavage fluid [21], indicating that CXCL16 on inflammatory cells plays a vital role in asthma development. We used flow cytometry to further verify the expression of CXCL16 of bronchoalveolar lavage fluid (BALF) on inflammatory cells. Figure 1E shows that the expression of CXCL16 was elevated and mainly expressed in the DC population after being induced by *Aspergillus*. To further confirm that CXCL16 is upregulated in mice challenged with *Aspergillus*, we obtained BMDCs by sorting using CD11c^+^ magnetic beads and differentiated them into mature DCs. We found that the CXCL16 levels in *Aspergillus-*induced BMDCs were higher than those from the control mice (Fig. 1F). The *P*-value associated with CXCL16 and phenotype of physician-diagnosed asthma is *P* = 2.33e−11 in UK Biobank 470k (v5) public (https://azphewas.com/phenotypeView/ba08a93f-501e-44e6-a332-98ce2f852279/51110ee5-9364-43ae-9cf8-1c63e99a4be6/glr). Taken together, these results reveal that CXCL16 is mainly expressed in dendritic cells and that its expression level was increased in *Aspergillus-*induced airway inflammation.

### Airway inflammation is reduced in *Aspergillus*-challenged CXCL16-knockout mice

**Fig. 2:**
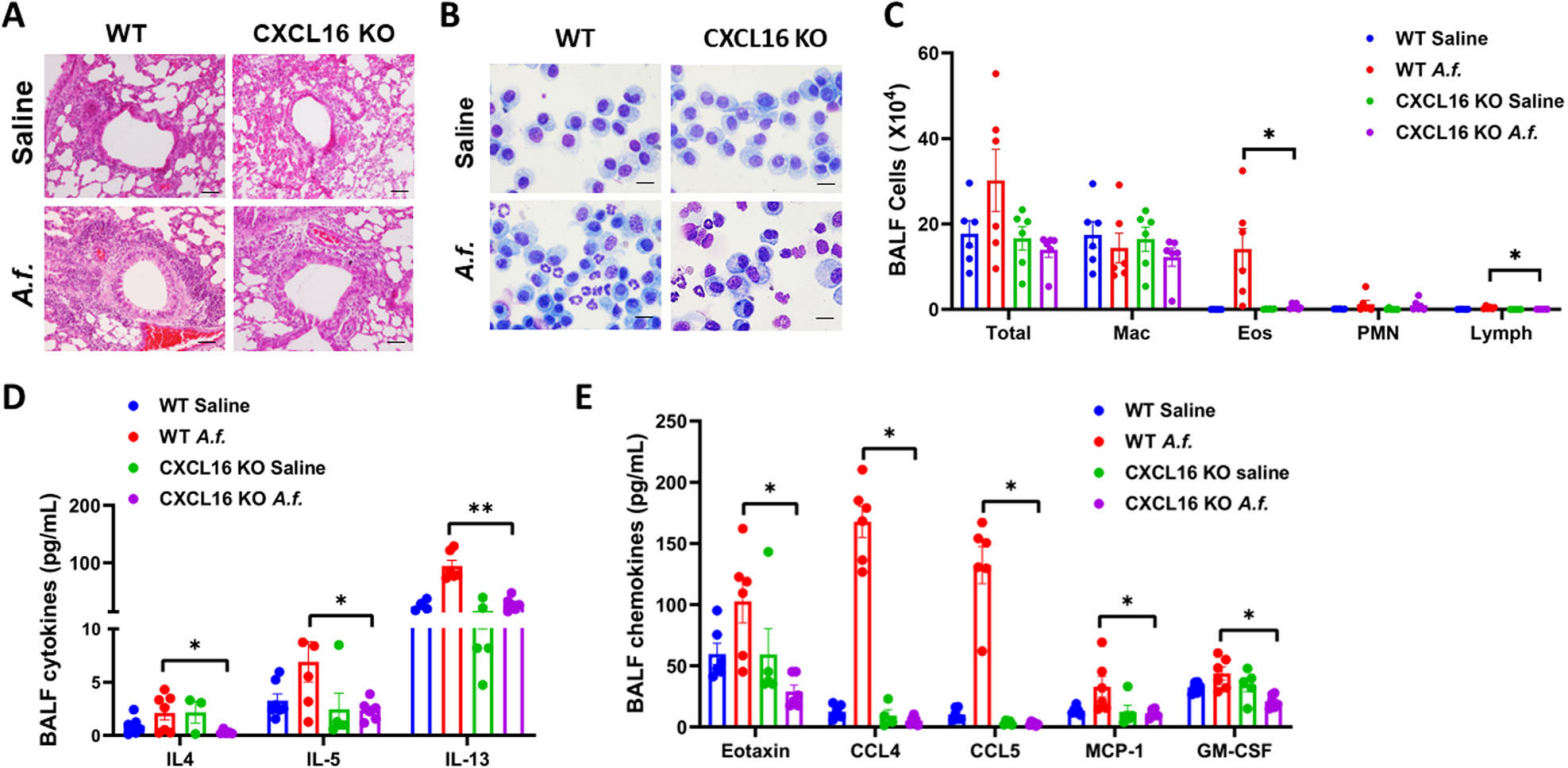
Airway inflammation was reduced in *Aspergillus*-challenged CXCL16-knockout mice. **A** Lung sections were stained with hematoxylin and eosin to show immune cell infiltration and hypertrophy of bronchial smooth muscle (×200 magnification and scale bar = 100 μm). **B** BALF cell Giemsa staining and (**C**) differential counts of wild-type and CXCL16-knockout mice were determined using light microscopy. PMN neutrophils, Eos eosinophils, Mac macrophages, Lymph lymphocyte (*n* = 6, with 400 total cells per sample). **D** Cytokine and (**E**) chemokine production in bronchoalveolar lavage was determined by multi-plex (*n* = 6). *P < 0.05, **P < 0.01 *Aspergillus*-challenged CXCL16-knockout mice versus wild-type mice. *A.f*. stands for *Aspergillus*.

To investigate the role of CXCL16 in *A.f*.-induced airway inflammation, CXCL16 knockout mice and wild-type mice were intratracheal instillation saline, or *Aspergillus fumigatus* extract for 3 weeks, and the airway inflammation was evaluated. The H&E staining showed that peribronchial and perivascular inflammation is suppressed in CXCL16-knockout (KO) mice induced by *Aspergillus* (Fig. 2A). Inflammatory cell infiltration in BALF and the inflammatory factors and chemokines in the BALF supernatant were evaluated. Giemsa staining revealed that the eosinophil counts in BALF of the CXCL16-knockout mice were reduced compared with those in the wild-type (WT) mice in response to the *Aspergillus* challenge (Fig. 2B, C). As reported, IL-4, IL-5, and IL-13 are proinflammatory cytokines; eotaxin is a pivotal chemokine crucial for eosinophil homing to the lungs of asthma patients [22]; and CCL4, CCL5, MCP-1, and GM-CSF recruit macrophages [23]. To investigate the levels of cytokines and chemokines in the CXCL16-knockout mice, we performed a multiplex cytokine assay. We found that the levels of Th2 cytokines IL-4, IL-5, and IL-13 in the CXCL16-knockout mouse BALF were decreased compared with those in the WT mice (Fig. 2D). The results of the levels of chemokines further confirmed that eotaxin, CCL4, CCL5, MCP-1, and GM-CSF in the BALF supernatant of the CXCL16-knockout mice were decreased (Fig. 2E). These data demonstrated that CXCL16 knockout reduces airway inflammation.

### Airway resistance and mucus production are reduced in *Aspergillus-*challenged CXCL16-knockout mice

**Fig. 3:**
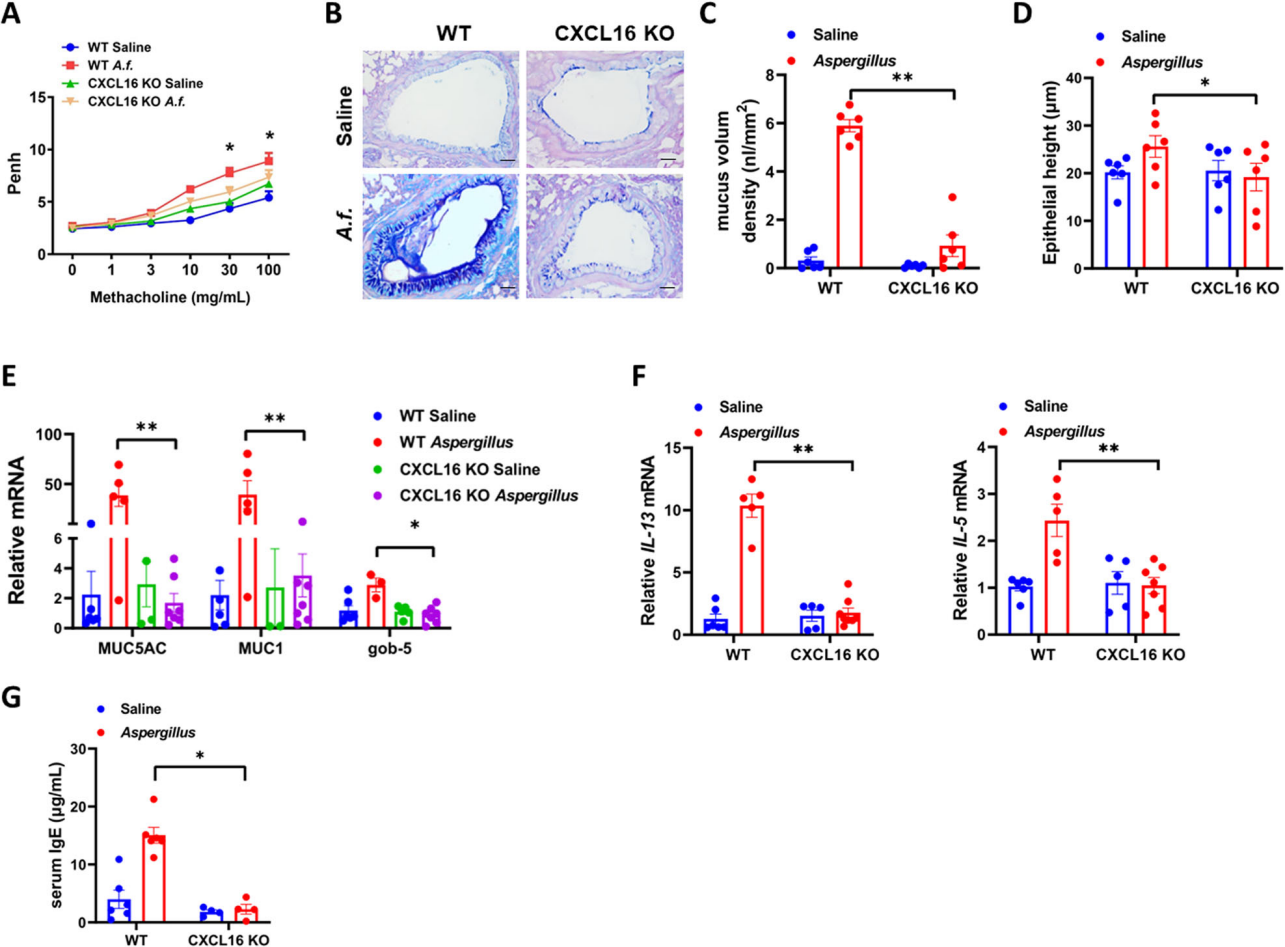
Airway resistance and mucus overproduction were reduced in *Aspergillus*-challenged CXCL16-knockout mice. **A** Bronchial hyperresponsiveness (BHR) to inhaled methacholine was measured by Penh in *Aspergillus*-challenged mice (*n* = 4). **B** Lung sections were stained with Alcian blue–periodic acid Schiff to visualize mucus oversecretion (×200 magnification and scale bar = 100 μm). **C** Mucus volume density and (**D**) epithelial height were determined using NIS-ELEMENTS quantitative automatic program (*n* = 6). **E**, **F** Quantitative analysis of Muc1, Muc5AC, Gob-5, IL-5, and IL-13 mRNA expression using real-time PCR (*n* = 5–6). **G** Serum IgE levels were determined by ELISA (*n* = 6). Data shown are mean ± SEMs. *P < 0.05, **P < 0.01 *Aspergillus*-challenged CXCL16-knockout mice versus wild-type mice. *A.f*. stands for *Aspergillus*.

In addition to chronic airway inflammation, airway hyperresponsiveness, and excessive mucus secretion are among the characteristics of asthma. To further investigate the role of CXCL16 in *Aspergillus-*induced asthma, we used CXCL16-knockout mice and WT littermates induced by *Aspergillus* to observe the airway hyperresponsiveness and overproduction of mucus. As expected, compared with that in the WT mice challenged with *Aspergillus*, airway resistance decreased in the CXCL16-knockout mice challenged with *Aspergillus* (Fig. 3A). The AB-PAS staining showed that overproduction of mucus (Fig. 3B, C) and goblet cell hyperplasia (Fig. 3D) in the CXCL16-knockout mice reduced after they were challenged with *Aspergillus*. In addition, the expression level of mucus-associated protein has confirmed these results in *Aspergillus*-challenged CXCL16-knockout and WT mice (Fig. 3E). Furthermore, the level of IL-13, which regulates mucus secretion, decreased in the CXCL16-knockout mice after the *A.f*. challenge, further confirming these results (Fig. 3F). Serum IgE results further revealed that inflammation response was decreased in the CXCL16-knockout mice challenged with *Aspergillus* (Fig. 3G). These results indicate that CXCL16 aggravates the pathogenesis of *Aspergillus*-induced asthma in mice.

### CXCL16^+^ dendritic cells have the ability to promote airway inflammation

**Fig. 4:**
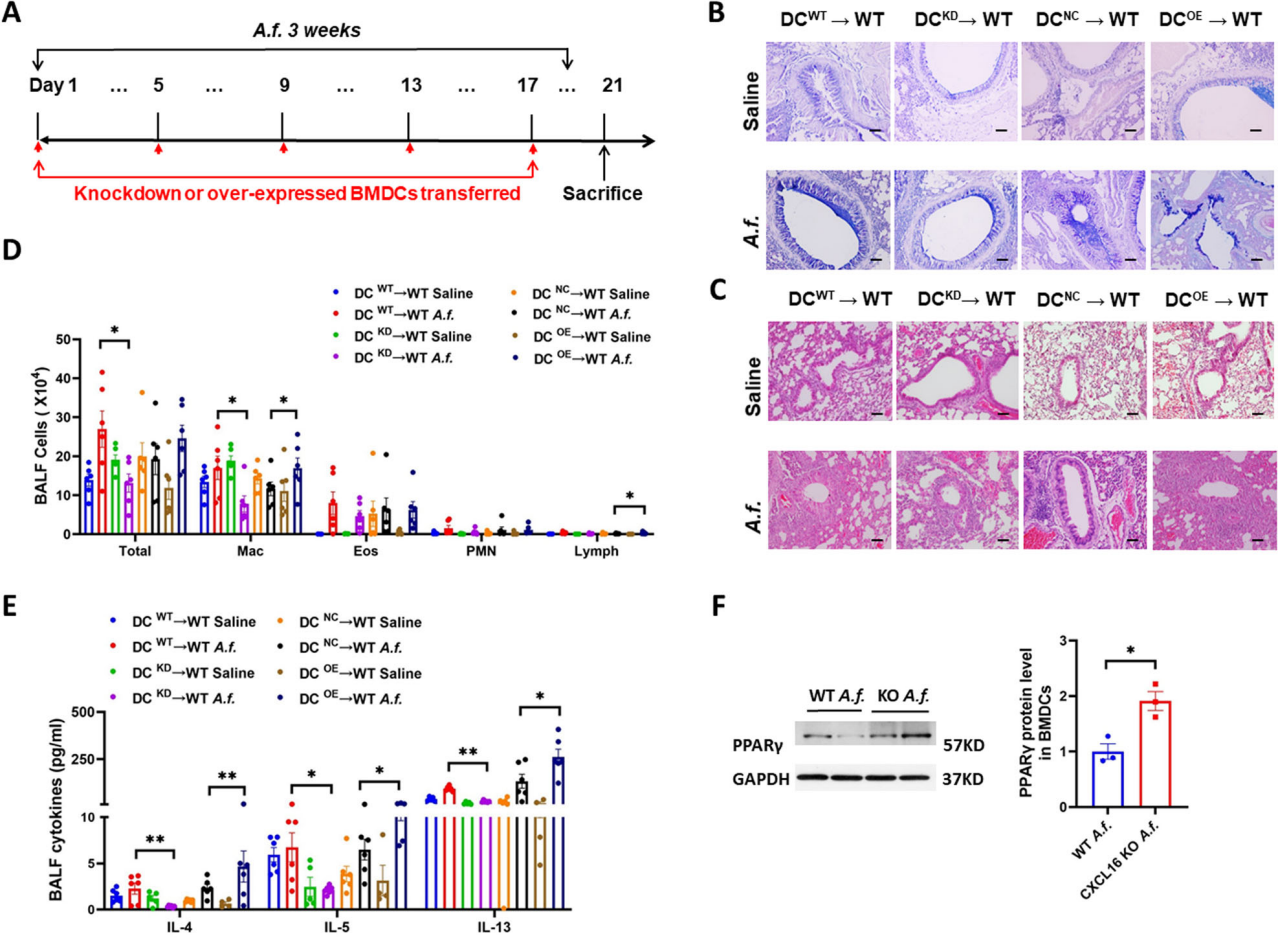
CXCL16 in dendritic cells has the ability to influence airway inflammation. **A** Wild-type recipient mice were administrated with CXCL16-knockdown donor of CD11c^+^ BMDCs or CXCL16-overexpressed CD11c^+^ BMDCs every 4 days followed by *Aspergillus* challenge every other day for 3 weeks. **B** Representative lung sections stained with Alcian blue–periodic acid Schiff (×200 magnification and scale bar = 100 μm). **C** Lung sections stained with hematoxylin and eosin (×200 magnification and scale bar = 100 μm). **D** Numbers of total BALF inflammatory cells and differential counts (*n* = 6). **E** Th2 cytokine production in bronchoalveolar lavage (*n* = 6). **F** The protein expression of PPARγ was detected by western blot (*n* = 3). *P < 0.05, **P < 0.01 *Aspergillus*-challenged CXCL16-knockdown donor mouse BMDCs versus wild-type donor mice, or CXCL16-overexpressed BMDCs versus wild-type donor mice. DC^WT^ → WT, wild-type donor mouse BMDCs adoptively transferred to wild-type mice; DC^KD^ → WT, CXCL16-knockdown donor mouse BMDCs adoptively transferred to wild-type mice; DC^NC^ → WT, CXCL16 negative control plasmid–transferred BMDCs adoptively transferred to wild-type mice; DC^OE^ → WT, CXCL16-overexpressed plasmid-transferred BMDCs adoptively transferred to wild-type mice. *A.f*. stands for *Aspergillus*.

After confirming the localization of CXCL16 on DCs and its high expression after *A.f*. stimulation, and CXCL16 knockout suppressed airway inflammation, we further explored the role of CXCL16^+^ DCs and CXCL16^−^ DCs on *Aspergillus*-challenged airway inflammation. We adoptively transferred *A.f*.-induced CXCL16-knockdown BMDCs or CXCL16-overexpressed BMDCs to wild-type mouse airways to evaluate the effect of CXCL16^+^ DC on airway inflammation. Figure 4A shows the process of adoptive transfer. We sorted the wild-type BMDCs using a CD11c^+^ separation kit, transfected with CXCL16-knockdown or CXCL16-overexpressed plasmid. After maturation, we adoptively transferred the DCs to wild-type mouse airways every 4 days and executed an asthma model with *Aspergillus*. Alcian blue–periodic acid Schiff (AB-PAS) staining showed that mucus overproduction was significantly suppressed in the CXCL16-knockdown BMDC-transferred mice and increased in the CXCL16-overexpressed BMDC-transferred mice (Fig. 4B). Hematoxylin and eosin (H&E) staining revealed that the inflammatory cell infiltration in the peribronchial tissue decreased in the CXCL16-knockdown BMDC-transferred mice and increased in the CXCL16-overexpressed BMDC-transferred mice (Fig. 4C). These results further confirmed inflammatory cell infiltration and cytokine detection of BALF. The inflammation cells in BALF (Fig. 4D) and the expression of Th2 cytokines (Fig. 4E) were decreased in the CXCL16-knockdown BMDC-transferred mice and increased in the CXCL16-overexpressed BMDC-transferred mice. These data demonstrated that CXCL16^+^ DCs aggravate *Aspergillus*-induced airway inflammation.

Previous studies have shown that PPARγ mediates anti-inflammatory effects of several immune cell types and induces CD4 dysfunction in BMDCs, and β-catenin signaling may limit the inflammatory response in the intestine, induce Tregs, and control Th17 response [24]. We hypothesized that PPARγ or β-catenin is involved in the anti-inflammatory effect of CXCL16 knockout BMDCs induced by *Aspergillus*. We used western blot to evaluate the protein expression level of PPARγ and β-catenin. We found that the protein expression level of PPARγ of CD11c^+^MHC II^hi^ DCs in the CXCL16-knockout DCs increased compared with that in the WT DCs after mice were challenged with *Aspergillus* (Fig. 4F). These data demonstrated that PPARγ signaling might be involved in CXCL16 knockout DCs suppress airway inflammation.

### CXCL16 knockout downregulates DC antigen processing and presentation

**Fig. 5:**
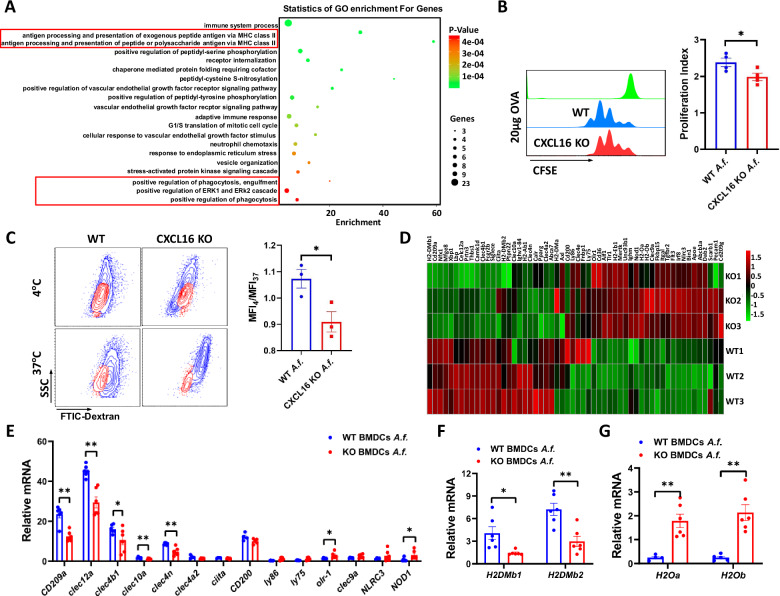
CXCL16 knockout suppressed DC antigen processing and presentation. **A** GO enrichment of biological process for downregulated differentially expressed genes in CXCL16-knockout mouse BMDCs versus wild-type mouse BMDCs after *Aspergillus* challenge (*n* = 3). **B** CD11c^+^ BMDCs from wild-type mice or CXCL16-knockout mice were pulsed with 20 μg OVA_323-339_ peptide and incubated at a 1:10 ratio with CFSE-labeled splenic CD4^+^ OT-II T cells for 4 days. OVA-specific proliferation is presented as a proliferation index (*n* = 4). **C** Phagocytosis of FITC-dextran by CD11c^+^ sorting BMDCs in vitro (*n* = 3). **D** Unsupervised clustering of genes involved in biological processes of antigen processing and presentation, MHC class II biosynthetic process, regulation of phagocytosis, phagocytosis, and recognition were analyzed (*n* = 3). **E** Real-time PCR was performed to detect downregulated differentially expressed PRR genes of *Aspergillus*-challenged CXCL16-knockout BMDCs and wild-type BMDCs (*n* = 3). **F** The mRNA level of H2-DM on *Aspergillus*-induced CXCL16-knockout mouse BMDCs or wild-type mouse BMDCs (*n* = 6). **G** The mRNA level of H2-Oa and H2-Ob in CXCL16-knockout BMDCs (*n* = 6). *P < 0.05, **P < 0.01 *Aspergillus*-challenged CXCL16-knockout mice versus wild-type mice. *A.f*. stands for *Aspergillus*.

Considering that the CXCL16^+^ DCs exerted a promoting role in airway inflammation, the CXCL16^−^ DCs inhibit airway inflammation. We further explore whether CXCL16 knockout in DCs suppressed DC’s function. To investigate the biological process behind CXCL16^−^ DCs suppressed airway inflammation after we induced CXCL16-knockout mouse BMDCs and WT mouse BMDCs with *Aspergillus*, we performed mRNA expression profiling of BMDCs using a GeneChip Mouse Transcriptome Array. Gene Ontology (GO) enrichment analysis of biological processes showed that compared with the WT mouse BMDCs challenged with *Aspergillus*, the enrichment of downregulated differentially expressed genes focused on antigen processing and presentation of MHC II and antigen phagocytosis (Fig. 5A). To confirm that CXCL16 knockout downregulates DC antigen processing and presentation, we evaluated the ability of CD11c^+^-sorted DCs to induce antigen-specific CD4^+^ T-cell proliferation. We found that CD11c^+^ BMDCs from the CXCL16-knockout mice suppressed T-cell proliferation (Fig. 5B). The RNA-sequencing data also suggested that CXCL16 knockout is involved in regulating the DC function of antigen phagocytosis. To clarify, we evaluated the phagocytose experiment of CXCL16 KO BMDCs and WT BMDCs with FITC-dextran in vitro. We found that the ability of CXCL16-knockout BMDCs to phagocytose FITC-dextran in vitro was suppressed compared with the WT BMDCs (Fig. 5C). All above data indicated that the function of antigen processing and presentation in CXCL16^−^ DCs is decreased.

These results demonstrated that CXCL16 knockout inhibited airway inflammation by suppressing the antigen processing and presentation function of DCs.

### CXCL16 suppress H2-DM and PRRs

To further investigate the mechanism behind how CXCL16 affects antigen presentation and antigen uptake by DCs, we further analyzed the mRNA expression profiles of CXCL16-knockout BMDCs and WT BMDCs induced by *Aspergillus*. Unsupervised clustering was performed on the differentially expressed genes from GO analysis involved in biological processes such as antigen processing and presentation, MHC class II biosynthesis, phagocytosis regulation, phagocytosis, and recognition. We found that these downregulated differentially expressed genes mainly included the MHC class II-related molecule H2-DM and pattern recognition receptors (PRRs) expressed on the surface of the cell membrane (Fig. 5D). We performed RT-PCR to verify the expression level of the differentially expressed genes with differential expression multiples > 1.5 and *P* < 0.05 in the clustering diagram. We found that the PRR mRNA levels of DC-SIGN (CD209a), DCAR (clec4b1), clec10a, clec12a, Dectin-2 (clec4n), and clec4a2 were decreased in CXCL16-knockout BMDCs induced by *Aspergillus* (Fig. 5E).

The processing and presentation of peptide segments after DC antigen uptake are dependent on MHC II molecules. It has been reported that the nonclassical MHC molecule H2-DM accounts for 50% of the MHC II epitopes and plays an important role in exchanging antigens with class II-associated invariant chain peptides (CLIPs). Compared with CLIP interaction with MHC II, DM binding of peptides to MHC molecules with higher affinity facilitates the presentation of antigen epitopes [25, 26]. In RNA sequencing, the top decreased expression gene in CXCL16 KO BMDCs is H2-DM. Thus, we hypothesized that CXCL16 KO suppressed the expression of H2-DM. To clarify this, we evaluated the mRNA level of H2-DM in *Aspergillus*-induced CXCL16-knockout BMDCs and WT BMDCs. We found that the H2-DM mRNA levels of H2-DMb1 and H2-DMb2 were decreased in CXCL16-knockout BMDCs induced by *Aspergillus* (Fig. 5F). It has been reported that another relatively nonpolymorphic MHC II molecule, H2-O/H2-DO, has an inhibitory function for H2-DM [26]. The molecule H2-O consists of Oα (encoded by H2-Oa gene) and Oβ protein (encoded by H2-Ob). We found that the expression of H2-Oa and H2-Ob was increased in CXCL16 KO BMDCs in RNA sequencing, and we performed RT-PCR to further confirm it. The results show that the mRNA levels of H2-Oa and H2-Ob were increased in the CXCL16-knockout BMDCs compared with the WT BMDCs (Fig. 5G). These data demonstrated that CXCL16 knockout downregulates the DC function of antigen processing and presentation by disrupting the balance of H2-DM/H2-DO.

The above results indicate that CXCL16 knockout downregulated the biological functions of DCs antigen processing and presentation by disrupting the balance of H2-DM/H2-DO, but whether CXCL16 deficiency affects the phenotype of DCs themselves is unknown.

### CXCL16 knockout promotes the dendritic cell differentiation

**Fig. 6:**
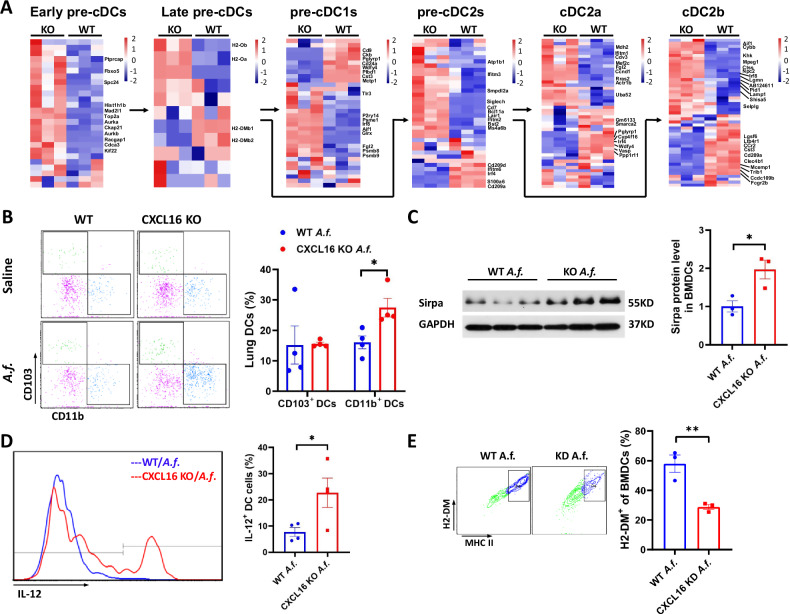
CXCL16 deficiency regulates dendritic cell differentiation. **A** Supervised clustering of genes involved in Early pre-cDCs, Late pre-cDCs, pre-cDC1s, pre-cDC2s, cDC2a, and cDC2b genes and their expression in CXCL16 knockout BMDCs and wild-type BMDCs. **B** Percentage of lung CD103^+^ DCs and CD11b^+^ DC subsets quantified by flow cytometry (*n* = 4). **C** The protein expression level of Sirpa in BMDCs detected by western blot (*n* = 3). **D** Intracellular secretion of IL-12 by lung CD11c^+^/MHC II^hi^/Siglec-F^−^ DCs from *Aspergillus*-challenged wild-type and CXCL16-knockout mice (*n* = 4). **E** Flow cytometry detected H2-DM on CXCL16-knockdown BMDCs. The percentage of H2-DM^+^ BMDCs of CD11b^+^/CD11c^+^/MHC II^hi^ BMDCs in *Aspergillus*-induced CXCL16-knockdown BMDCs and WT BMDCs (*n* = 3). Data shown are mean ± SEMs. *P < 0.05, **P < 0.01 *Aspergillus*-challenged CXCL16-knockout mice versus wild-type mice. *Aspergillus*-induced CXCL16 knockdown BMDCs versus WT BMDCs. KD knockdown, WT wild-type. *A.f*. stands for *Aspergillus*.

We noticed that the molecular H2-DM/H2-DO is also a key regulating factor in early pre-cDCs differentiated from late pre-cDCs. To explore whether CXCL16 knockout in DCs affects the differentiation phenotype of DCs themselves, the RNAseq data was compared with the list of the genes previously identified as Early pre-cDCs, Late pre-cDCs, pre-cDC1s, pre-cDC2s, cDC2a and cDC2b [27]. As shown in Fig. 6A, we observed that in CXCL16 deficient DCs, some characteristic genes (Cd80, H2-DMb1, H2-DMb2) were lost among early pre-cDCs cluster differentiated into late pre-cDCs cluster while some key regulators (Atp1b1, Siglech, Lair1, Ifitm2, Ifitm3, Bst2, Smpdl3a, Bcl11a, Cd7, Ms4a6b) were overexpression in late pre-cDC2 cluster differentiated into the pre-cDC2 cluster, meanwhile, the cluster of cDC2a and cDC2b was imbalanced during the differentiation of CXCL16 deficient cDC2s. Some cDC2b related genes are down-regulated (Cdc109b, Cd209a, Ccr2, Trib1, Clec4b1, Ltb4r1, Cst3, Fcgr2b, Igsf6, Mcemp1, Clec12a), while cDC2a overexpressed genes include Ccnd1, Ifitm1, Mef2c, Rras2, Mdh2, Fgl2 and other key factors. According to our data, these results indicated that CXCL16 knockout led to the tendency of late pre-cDC cells to differentiate into cDC2, and CXCL16 knockout suppressed cDC2 further differentiated into cDC2b. Considering that cDC2b is mainly charged with antigen presentation and plays a pro-inflammation role, we hypothesize that CXCL16 knockout suppressed cDC2b differentiation and promoted cDC2a differentiation.

To further confirm that CXCL16 knockout in DCs affects the differentiation of DCs, we performed digestion of the DCs in lung tissue cells for flow cytometry. The total amount of DCs is evaluated by flow cytometry. We found that there was no significant difference in the total number of CD11c^+^/Siglec F^−^/MHC II^hi^ DCs between the *Aspergillus*-challenged CXCL16-deficient and WT mice (Supplemental Fig. 5A). To explore whether CXCL16 knockout alters the expression of costimulatory cell surface molecules in response to *Aspergillus*-induced asthma, we detected the expression of CD80 and CD86 of the DCs in lung tissue cells for flow cytometry. We found that there was a decreased number of CD80 on CD11c^+^/Siglec F^−^/MHC II^hi^ DCs and no differences in the cell surface expression of the costimulatory molecule of CD86 on CD11c^+^/Siglec F^−^/MHC II^hi^ DCs between the *Aspergillus*-challenged CXCL16-knockout and WT mice (Supplemental Fig. 5B).

Next, we try to figure out whether CXCL16 knockout in DCs alters the DCs subgroup. As shown in Fig. 6B, the number of CD11b^+^CD103^−^ DCs (cDC2 DCs) is increased among CD11c^+^/Siglec F^−^/MHC II^hi^ DCs in the *Aspergillu*s-induced CXCL16-knockout mice, compared with the WT mice. To further confirm that CXCL16 knockout mainly increased the number of cDC2 DCs, the protein expression level of CD172a (Sirpα) was evaluated because CD172a separates cDC1 DCs from cDC2 DCs and plays a pivotal role in regulating the differentiation of DC2 [28]. As shown in Fig. 6C, the protein expression level of Sirpα further confirmed that cDC2 DC subsets increased and the protein level of Sirpα increased in the CXCL16-knockout cDC2 DCs compared with the WT cDC2 DCs.

To test and verify that CXCL16 knockout promotes cDC2 differentiation to cDC2a, we evaluated the cDC2a ability of cytokines production of CXCL16^+^ DCs and CXCL16^−^ DCs. Intracellular secretion of IL-12 by lung CD11c^+^/Siglec-F^−^/MHC II^hi^ DCs was evaluated by flow cytometry analysis in vivo. The flow cytometry results showed that the secretion of IL-12 by DCs increased in the *Aspergillus*-challenged CXCL16-knockout mice (Fig. 6D). The above results indicate that CXCL16 deficiency promoted the cDC2 differentiation.

All the data indicated that CXCL16 knockout suppressed H2-DM molecular mediated cDC2b differentiation to reduce dendritic cell antigen processing and presentation. To further confirm the effect of CXCL16 on the H2-DM molecule, we transfected a CXCL16-knockdown plasmid to WT BMDCs and evaluated the expression level of H2-DM by flow cytometry. We found that the H2-DM expression level was decreased in the CXCL16-knockdown plasmid-transfected BMDCs (Fig. 6E).

## Discussion

The present study demonstrated that CXCL16 knockout inhibits DC antigen processing and presentation by downregulating H2-DM, thereby inhibiting Th2 airway inflammation induced by *A. fumigatus*.

The location of CXCL16 in types of cells that participate in the development of asthma airway inflammation has been reported as controversial. In asthma patients, CXCL16 is expressed in airway epithelial cells [29] and alveolar macrophages [21]. In animal models, CXCL16 is expressed in endothelial cells [30] and various immune cells, including DCs [16] and B cells [31]. Our results demonstrated that CXCL16 is mainly expressed in DCs in asthma patients and asthma mouse models by scRNA-seq data. We further identified CXCL16 expressed increased in DCs of the asthma lung by flow cytometry and western blot assay. Adoptive transfer experiments further confirmed that the DCs expressed CXCL16, but no other type of cells expressed CXCL16 exhibited a promoting role in asthma airway inflammation. These data provided strong support for the location of CXCL16 in DCs in asthma.

Recent studies and our experiments have revealed that CXCL16 has a promoting effect on airway inflammation. It has been reported that compared with the control saline challenge, intranasal stimulation of mice with IL-25, IL-33, or OVA increased the concentration of CXCL16 in the lungs [32]. The expression peaks of CXCL16 induced by IL-33, IL-25, or OVA were, on average, observed on day 25. When an anti-CXCL16 antibody was used to block CXCL16, there was a significant decrease in the number of ILC2 in BALF and mediastinal lymph nodes compared with the control antibody treatment of WT mice after HDM treatment. These results are similar to ours. In the present study, in *Aspergillus*-induced airway inflammation in mice, the expression of CXCL16 was most significantly elevated. After CXCL16 knockout, the inflammatory cell infiltration around the airway was reduced, and the secretion of airway mucus and the expression of mucus secretion-related proteins were reduced. According to our results, CXCL16 plays a pro-inflammatory role in *Aspergillus*-induced airway inflammation, and inhibition of CXCL16 suppresses Th2 inflammation.

It is reported that ERKs were activated in BMDCs in antigen processing at an early stage [33], ERK activation prevents T cell expansion, while ERK inhibition increases T response [34]. The results of RNA sequencing indicated that the ERK1/2 cascade in CXCL16 KO BMDCs is downregulated. These results are consistent with the literature reports, suggesting that the ERK pathway might be involved in the antigen processing and presentation of CXCL16^−^ DCs. We used western blot assay to verify the expression of p-ERK1/2 and ERK in both WT and CXCL16 KO BMDCs. We found that the phosphorylation of ERK is decreased in CXCL16 knockout BMDCs (Supplemental Fig. 3). These data suggested that the ERK pathway might be involved in CXCL16 knockout suppressed antigen processing and presentation of DCs.

Traditionally, it has been reported that DCs encompass two major subsets: CD103^+^CD11b^−^ (cDC1) and CD11b^+^CD103^−^ (cDC2) cells. Recently, the cDC2 is considered a heterogeneous population derived from different progenitor cell populations. It has been found that it can be classified according to gene characteristics and functions. In mouse spleen, cDC2 can be subdivided into two major subgroups, notch-dependent ESAM cDC2a and Clec12A cDC2b [35]. The cDC2a seems to have a higher ability to produce cytokines and play an anti-inflammatory role in inflammatory disease, while cDC2b has a higher ability of antigen presentation to T cells and plays a pro-inflammatory role in vivo [36]. Our data demonstrated that CXCL16 knockout promotes the differentiation of cDC2 towards cDC2a and reduces cDC2 differentiation towards cDC2b. The RNAseq results and antigen processing and presentation function of DCs, as well as the IL-12 production results support this hypothesis.

Notably, H2-DM is a molecular chaperon that releases endogenous class II-associated invariant chain peptide from MHC class II αβ dimers to allow the foreign peptide to assemble into the MHC II groove for surface presentation [25]. In H2-DM knockout mice, CLIP cannot be efficiently exchanged with other peptides, limiting MHC II antigen diversity [37]. Cystatin C downregulates the MHC II chaperon H2-DM of DCs, resulting in a decrease in MHC II peptide presentation and T-cell proliferation [38]. Thus, it is also possible that CXCL16 deficiency affects H2-DM. Our RT-PCR and cytometry experiments indicated that CXCL16 knockdown suppresses the MHC II antigen processing and presentation through the suppression of H2-DM. Another relatively nonpolymorphic MHC II molecule, H2-O/H2-DO, has an inhibition function for H2-DM [39]. H2-DM and H2-O in mice play an important role in the CLIP exchange with endosomal antigens [40]. The imbalance ratio of H2-DM and H2-O is related to MHC II antigen processing [41]. We performed RT-PCR to assess the expression of H2-Oa and H2-Ob. The results showed that the mRNA levels of H2-Oa and H2-Ob were increased in the CXCL16-knockout BMDCs compared with the WT BMDCs. This indicates that CXCL16 knockout may affect the interaction between H2-O and H2-DM, but the underlying mechanism needs to be further explored.

Although the exact signaling pathway triggered by CXCL16 in DCs is not clear, inhibition of CXCL16 in DCs can reduce the antigen presentation of DCs and inhibit airway inflammation. Recently, an increasing number of population cohorts have reported that CXCL16 polymorphism is associated with the efficacy of drugs to treat inflammation. For example, the CXCL16 T123V181 haplotype is associated with an increased risk of sepsis morbidity and a higher MOD score [42]. The CXCL16 rs2277680 GG + GA genotype is associated with the effectiveness of metformin versus placebo in overweight patients with knee osteoarthritis [43]. The genotype of CXCL16 rs8071286 locus significantly correlates with the serum levels of corresponding cytokines in critical cases of COVID-19 [44]. Therefore, further asthma patient cohort research is required to investigate whether CXCL16 polymorphism can lead to DC dysfunction and alleviate asthma airway inflammation. Targeted drugs and inhibitors that inhibit CXCL16 will be a very meaningful treatment approach for CXCL16 wild-type genotype individuals who suffer from asthma.

The limitation of our study was that we were not able to further elucidate the signaling pathways and mechanisms by which CXCL16 affects H2-DM.

The scavenger receptor is expressed on DCs and regulates antigen presentation and T priming. As such, the present study demonstrated that CXCL16 knockout played a protective role in *Aspergillus* fumigatus-induced airway inflammation. CXCL16 knockout in DCs inhibits antigen processing and presentation by suppressing the expression of the MHC class II molecule H2-DM, suggesting that inhibition of CXCL16 can be a potential therapy for asthma. Our results highlight the vital role of the CXCL16 in dendritic cells in antigen processing and presentation in asthma airway inflammation. In the future, the correlation between polymorphisms in CXCL16 patients and the degree of asthma disease in asthma patients will be evaluated, and the mechanism of how CXCL16 affects MHC II will be further studied.

## Materials and methods

### Mice

Littermate wild-type (WT) mice, CXCL16^**−/−**^ mice (Cyagen, Suzhou, China), and OT-II TCR transgenic mice (strain B6.Cg-Tg (TcraTcrb) 425Cbn/J; Cavins Laboratory Animal, Changzhou, China) aged 9–10 weeks were used for experiments. The mice were bred at the Experimental Animal Center of Beijing Institute of Heart, Lung and Blood Vessel Diseases. All animal model experiments were conducted under isoflurane anesthesia. Animals were euthanized by pentobarbital sodium anesthesia. All animal experiments were performed and approved by the animal ethics committee from the Experimental Animal Center of Beijing Institute of Heart, Lung and Blood Vessel Diseases (Approval No. AZ2024LA022).

### Model of *Aspergillus* challenge

The mice were intratracheally instilled with 5 μL *Aspergillus* extract (Hollister-Stier Laboratories, Spokane, WA) in a total volume of 20 μL under isoflurane anesthesia every other day for 3 weeks, as previously described [45].

In adoptive transfer experiments, CD11c^+^ BMDCs from WT mice or CXCL16-knockout mice pulsed with *Aspergillus* or saline were adoptively transferred to WT mice that were subsequently challenged by *Aspergillus*.

In overexpression adoptive transfer experiments, BMDCs from WT mice transfected with control plasmid or CXCL16-overexpressing plasmid were intratracheally instilled in WT mice every fourth day during the *Aspergillus-*challenge phase.

### Assessment of airway inflammation

At 24 h after the final challenge, the lung lobe was placed in fresh 10% formalin for subsequent periodic acid-Schiff (PAS) and hematoxylin & eosin (HE) staining. Mucus volume density and epithelium height were determined using the NIS-ELEMENTS quantitative automatic program (Nikon, Japan). The expression of CXCL16 was detected by immunohistochemistry staining and captured under a ×200 magnification optical microscope. The total bronchoalveolar lavage fluid (BALF) was collected, and the supernatant was stored at −80 °C for subsequent quantification of cytokines. The remaining cell pellet was resuspended and analyzed for differential cell counts in cytospin (Thermo cytospin4), followed by staining with Wright-Giemsa. Neutrophils, macrophages, and eosinophils were quantified via oil microscopy at a magnification of ×1000, and the average number of each cell type per high-power field was determined after counting a total of 400 cells.

### Assessment of airway hyperresponsiveness

Bronchial hyperresponsiveness to different doses of methacholine (0, 3, 10, 30, and 100 mg/mL) was measured via invasive plethysmography (Buxco, Wilmington, NC) as described previously.

### Multi-Plex and ELISA measurements

Protein levels of cytokines and chemokines in BALF were analyzed using a Luminex multiplex mice cytokine assay (Bio-Rad, Hercules, CA) in accordance with the manufacturer’s instructions. The data were analyzed using Bio-Plex Manager software (Bio-Rad^TM^ 200 System). Serum IgE was detected using a commercial ELISA kit (Abcam).

### RNA extraction and real-time PCR

Total RNA was obtained from the lungs using TRIzol (Invitrogen, Carlsbad, CA) in accordance with the manufacturer’s directions. A total of 2 μg RNA was reverse-transcribed into cDNA with M-MLV and random primer (Promega). Specific oligonucleotide primers were added to the buffer along with a 2 μL reverse-transcribed cDNA sample. The cDNA was amplified using the following cycling parameters. The mixture was first incubated for 4 min at 94 °C and then cycled 40 times at 94 °C for 30 s, 60 °C for 30 s, and elongated at 72 °C for 30 s. An iQ5 system (Bio-Rad) with SYBR Green I (Takara, Shiga, Japan) was used for real-time quantitative PCR analysis. GAPDH was used as an internal control. The level of mRNA was normalized to GAPDH expression, and the results were analyzed by the 2^−ΔΔCt^ method (Supplementary Table 1).

### BMDC and plasmid transfection

BMDCs were obtained from the tibial and femoral bone marrow of C57BL/6 mice. We collected bone marrow suspensions and filtered out small fragments and muscle tissue using a 70-μm nylon cell strainer. After centrifugation and washing with PBS, the cells were resuspended using RPMI 1640 culture medium containing 10% FBS with GM-CSF (40 ng/mL) and IL-4 (10 ng/mL). Half of the medium was replaced with a fresh medium every other day. On day 7 of cultivation, the semi-suspended cells were blown off, centrifuged, and resuspended in a 1640 culture medium containing 10% FBS with GM-CSF and IL-4. The cells were seeded onto a six-well plate and stimulated with LPS (100 ng/mL) for 24 h to obtain mature DCs before preparing for the next experiment. The mature BMDCs were exposed to *Aspergillus* (10 µg/mL) for 24 h for the next experiment.

Transfection with an overexpression plasmid and a knockdown plasmid was performed using the DCs electroporation program provided by the manufacturer (Lonza Walkersville Inc., Walkersville, MD). BMDCs were transfected in the Lonza Shuttle, using the P3 Primary Cell 4D X Kit (Lonza Walkersville Inc., Walkersville, MD). Briefly, DCs were harvested from six-well plates and washed twice in PBS. The cell suspension of 2 × 10^5^ cells was combined with plasmid DNA (2 μg) or siRNA (100 nM). Immediately after electroporation, the cells were transferred to RPMI 1640 medium supplemented with 10% FBS, penicillin, streptomycin, and l-glutamine in addition to GM-CSF and IL-4. Electroporation efficiency was assessed by flow cytometry at 2–4 h after electroporation using the GFP transfection indicator.

### Microarray analysis and bioinformatics analysis

For Affymetrix microarray profiling, the total RNA was isolated from BMDCs using TRIzol reagent (Invitrogen, Carlsbad, Canada) and purified with an RNeasy Mini Kit (Qiagen, Hilden, Germany) in accordance with the manufacturer’s protocol, including a DNase digestion treatment. The amount and quality of RNA were determined by a UV–Vis Spectrophotometer (Thermo, NanoDrop 2000, USA) at the absorbance of 260 nm. The mRNA expression was measured using GeneChip Mouse Transcriptome Array 1.0 (MTA 1.0) (Affymetrix Gene Chip, Santa Clara, CA), which contained 65,957 gene-level probe sets. The microarray analysis was performed by Affymetrix Expression Console Software (version 1.2.1). Raw data (CEL files) were normalized at the transcript level using a robust multi-array average method (RMA workflow).

For the microarray data analysis, differentially expressed genes were identified based on the Lima package of R. The differentially expressed genes were considered up- or downregulated at *P* < 0.05. Genes with similar expression patterns often facilitate overlapping functions. Accordingly, cluster analysis of gene expression patterns was analyzed by Cluster and Java Treeview software. Gene ontology (GO) analysis was applied to analyze the main functions of the differentially expressed genes according to the Gene Ontology project. Pathway analysis was used to identify the significant pathways of the differentially expressed genes according to the Kyoto Encyclopedia of Genes and Genomes (KEGG) database. Fisher’s exact test was performed to select the significant GO term and pathway, and the threshold of significance was set at *P* < 0.05.

### Single-cell RNA-sequencing analysis

The human scRNA-seq analyses were performed using lung cell profiles in the Lung Cell Atlas (https://asthma.cellgeni.sanger.ac.uk/) from https://lungcellatlas.org [46]. The publicly available datasets used were pre-annotated. The mouse scRNA-seq data of OVA-induced asthma lung were from #CRA004586 in the BIG submission Portal (BIG Sub) OMIX repository (https://ngdc.cncb.ac.cn/gsub/).

### Western blot analysis

BMDCs or lung tissue were lysed with a cell-lysis buffer (Cell Signaling Technology) containing a protease inhibitor cocktail (Calbiochem). Protein concentration was measured using a BCA assay (Thermo Scientific). Antibody against CXCL16 (# PA5-81370, 1:1000 dilution) was obtained from Thermo Fisher. Antibody against Sirpα (AF0253, 1:1000 dilution) was obtained from Affinity. Antibodies against PPARγ (2435s, 1:1000 dilution) were obtained from Cell Signaling Technology. Antibody against GAPDH (YM3029, 1:20,000 dilution) was obtained from ImmunoWay.

### Flow cytometry

CXCL16 expression in BALF cells was examined using the following antibodies: CD45 percy-cy5.5 (# 550994, BD), CD11c AF488 (#53-0114-82. eBioscience), F4/80 PE (# 123110, Biolegend), CD11b BV650 (#563402, BD), Gr-1 PE-Cy7 (# 60-5931-U100, TONBO), Siglec-F PE-CF594 (#562757, BD), CD3 APC-cy7 (#25-0032-U025, TONBO), CXCL16 (# AF503, R&D), and donkey anti-goat BV421 (#ab175664, Abcam).

Lung cells were isolated by enzymatic digestion with collagenase IA (1.6 mg/mL, Sigma) incubated at 37 °C for 30 min with agitation. Mouse total DCs subsets were identified and characterized using the following antibodies: CD11c AF488 (# 53-0114-82, eBioscience), Siglec-F PE-CF594 (#562757, BD); MHC II APC-EF780 (#47-5321-82, Invitrogen); CD45 PerCP-Cy5.5 (# 550994, BD); and CD11b APC (#17-0112-82, eBioscience). Mouse DCs maturation was detected by CD11c PerCP-Cy5.5 (#560584, BD); Siglec-F PE-CF594 (#562757, BD); MHC II PE (#12-5321-82, eBioscience); CD80 APC (#16-10A1, eBioscience); and CD86 PE-cy7 (#25-0862-82, eBioscience). Lung DCs subsets were detected by CD11c PerCP-Cy5.5 (#560584, BD), Siglec-F PE-CF594 (#562757, BD), MHC II PE (#12-5321-82, eBioscience), CD103 APC (#17-1031-82, eBioscience), and CD11b AF488 (eBioscience). Intracellular secretion of IL-12 by lung CD11b^+^ DCs subsets was detected using CD11c PerCP-Cy5.5 (#560584, BD), Siglec-F PE-CF594 (#562757, BD), MHC II APC- EF780 (#47-5321-82, Invitrogen), CD103 APC (#17-1031-82, eBioscience), CD11b BV650 (#563402, BD), and IL-12 PE (#505203, BioLegend).

The expression of the H2-DM on BMDCs was examined: CD11c AF488 (# 53-0114-82, eBioscience), MHC II APC-EF780 (#47-5321-82, Invitrogen), purified rat anti-mouse H2-DM/H2-M (#552405, BD), and goat anti-rat AF647 (#ab150167, Abcam).

Data were acquired on LSRFortessa (BD Biosciences, USA) and analyzed by FlowJo 7.6 software.

### DCs antigen uptake

In vitro, BMDCs were incubated with 1 mg/mL FITC-dextran (Sigma) for 20 min at 4 or 37 °C. The phagocytosis/internalization was calculated as the change in MFI between the cell samples incubated at 37 and 4 °C.

### Antigen processing and presentation

The ability of DCs to induce antigen-specific T-cell proliferation was assessed using carboxyfluorescein diacetate succinimidyl ester (CFSE)-labeled splenic CD4^+^ T cells from OT-II mice. OT-II mice expressing MHC II-restricted DO11.10T cell receptor that recognizes OVA_323-339_ peptide. BMDCs from WT mice or CXCL16-knockout mice were pulsed for 24 h with 5 or 20 µg OVA_323-339_ peptide (Sigma) or PBS as a control. CD11c^+^ cells were isolated by a CD11c^+^ bead-positive selection kit (MACS Miltenyi Biotec). Naïve CD4^+^ T cells were purified from the spleens of OT-II mice by a CD4^+^ bead negative selection kit (MACS Miltenyi Biotec) and were labeled with 5 µM CFSE (ThermoFisher Scientific) at 37 °C for 30 min. The cells were mixed at a ratio of 1:10 (2 × 10^4^ CD11c^+^ DCs, 2 × 10^5^ OVA peptide-specific CD4^+^ OT-II cells) and incubated for 4 days. The proliferation index analyzed by FlowJo was used as an indicator of cell division.

### Statistical analysis

Data are presented as the mean ± standard error of the mean (SEM). Statistical differences between experimental groups were assessed by one-way analysis of variance (ANOVA) using GraphPad Prism (Version 5, GraphPad Software Inc., San Diego, CA) and described in each figure legend. In animal experiments, mice of the same age and similar weight were randomly divided into different groups using a double-blind method. Researchers conducting animal experiments do not know the animal grouping, while statistical analysis researchers know the grouping. Before conducting formal experiments, conduct preliminary experiments to estimate the similar variation of each group/treatment. The formal experiment involves eight mice per group to ensure sufficient adequate power. Experimental data outside of mean ± 3 SD were excluded. *P*-values < 0.05 were considered significant.

## Supplementary information

Supplemental material

Supplemental table 1

## Data Availability

The BMDC RNA-seq data were uploaded to the BIG submission Portal (BIG Sub) OMIX repository (OMIX accession number #OMIX005155; https://ngdc.cncb.ac.cn/gsub/).
