# Supplementary material for: CXCL16 knockout inhibit asthma airway inflammation by suppressing H2-DM molecular mediated antigen presentation
Source: Cell Death Discov. 2025 Mar 6;11:90. doi: 10.1038/s41420-025-02371-6 (PMC11885808; doi:10.1038/s41420-025-02371-6)
Supplement: Supplementary file 1 — Supplemental material [file 41420_2025_2371_MOESM1_ESM.pdf]

Supplemental figure 1

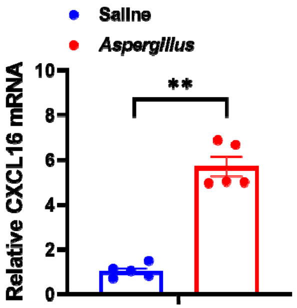


Supplemental figure 1. Quantitative analysis of CXCL16 mRNA expression. Quantitative analysis of CXCL16 mRNA expression using real-time PCR ($n$ = 5). Data shown are mean ± SEMs. **$P$ < 0.01 *Aspergillus*-challenged wild-type mice versus saline-challenged wild-type mice.

Supplemental figure 2.

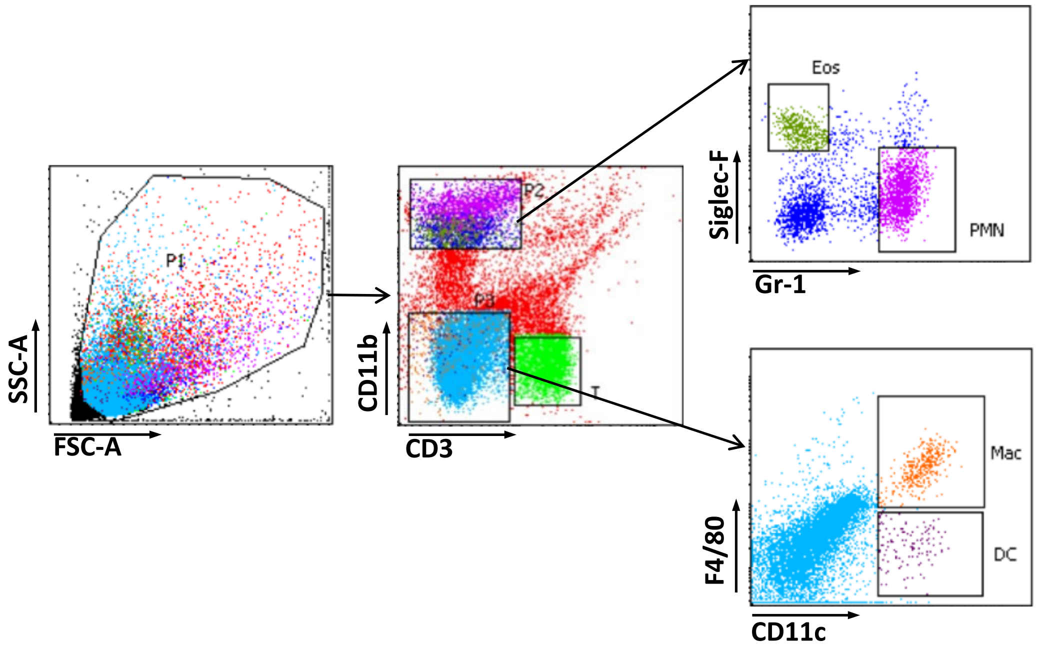


Supplemental figure 2. Gate for lung CD11c$^{+}$ subgroups. Gate for CXCL16$^{+}$ cell expression was assessed by using flow cytometry on inflammatory cells in bronchoalveolar lavage. CD11b$^{+}$/CD3$^{-}$/Siglec-F$^{+}$/Gr-1$^{-}$ (Eosinophils), CD11b$^{+}$/CD3$^{-}$/Siglec-F$^{-}$/Gr-1$^{+}$ (PMNs), CD11b$^{-}$/CD3$^{-}$/F4/80$^{+}$/CD11c$^{+}$ (Macrophages), CD11b$^{-}$/CD3$^{-}$/F4/80$^{-}$/CD11c$^{+}$ (Dendritic cells), and CD11b$^{-}$/CD3$^{+}$ (T) cells. PMN, neutrophils. Eos, eosinophils. DC, dendritic cells. Mac, macrophages.

Supplemental figure 3

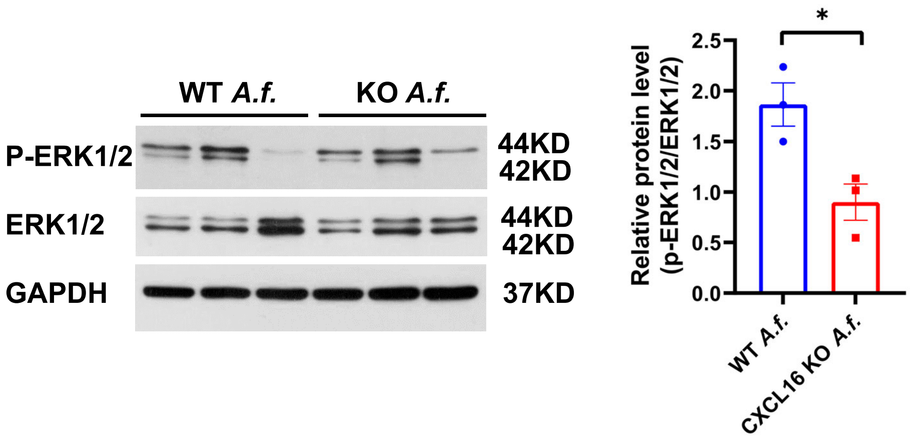


Supplemental figure 3. The protein expression of ERK1 and ERK2. The ERK pathway in *Aspergillus*-induced wild-type mouse BMDCs or CXCL16-knockdown BMDCs detected by western blot (n = 3). *P < 0.05 Aspergillus-challenged CXCL16-knockout mice versus wild-type mice. *A.f.* is stand for *Aspergillus*.

Supplemental figure 4.

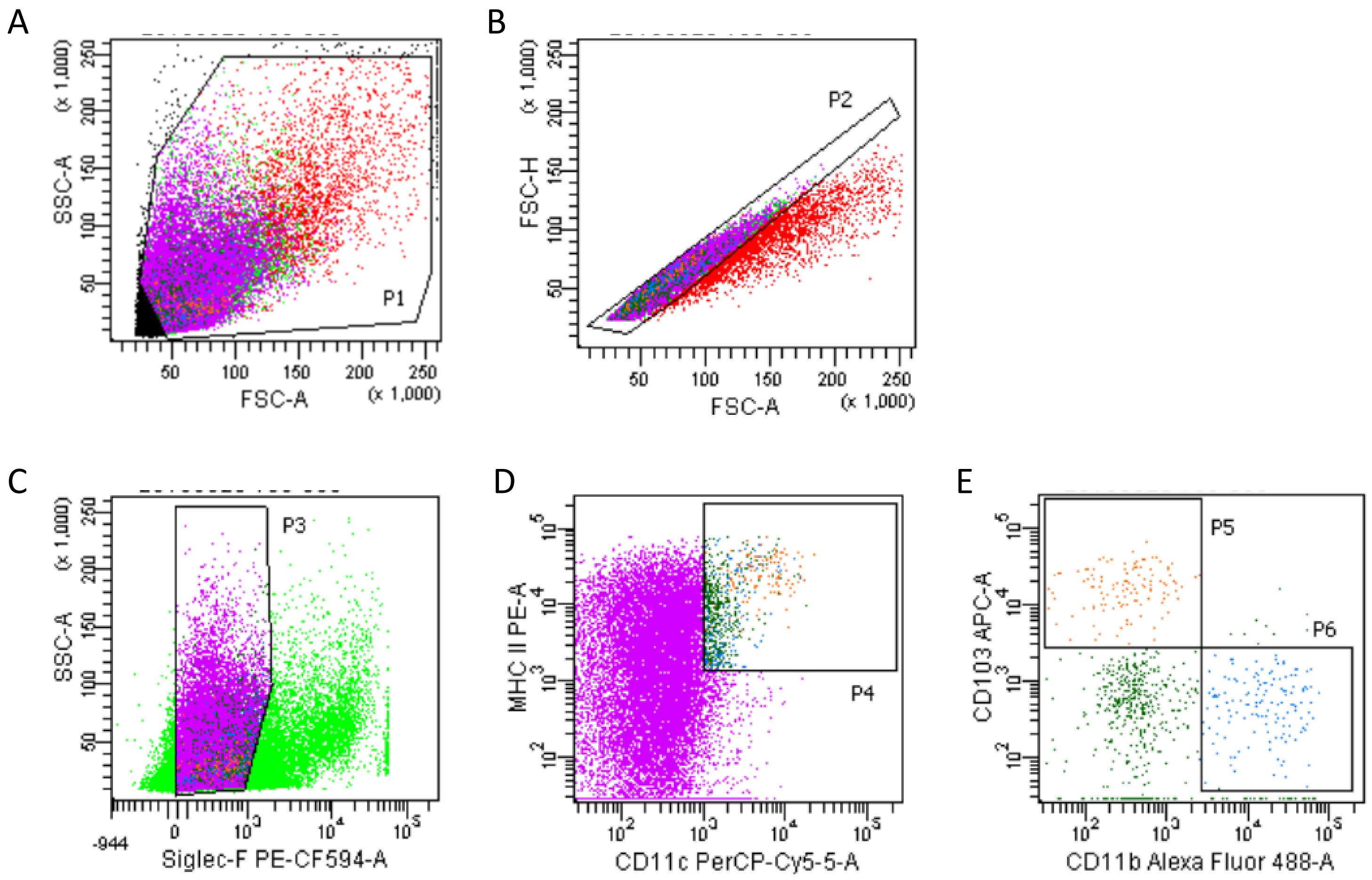


Supplemental figure 4. Gate for CD11b$^{+}$CD103$^{-}$/CD11b$^{-}$CD103$^{+}$ subgroups. Gate for cDC1 and cDC2 subsets by flow cytometry. (A) P1 for the lung single cells of CXCL16 KO or WT mice induced by *Aspergillus* for 21days. (B) P2 for deadhesion cells of P1. (C) P3 for Siglec-F$^{-}$ cells of P2. (D) P4 for CD11c$^{+}$MHC II $^{hi}$ cells of P3. (E) P5 for CD11b$^{-}$CD103$^{+}$ cells of P4, and P6 for CD11b$^{+}$CD103$^{-}$ cells of P4.

Supplemental figure 5

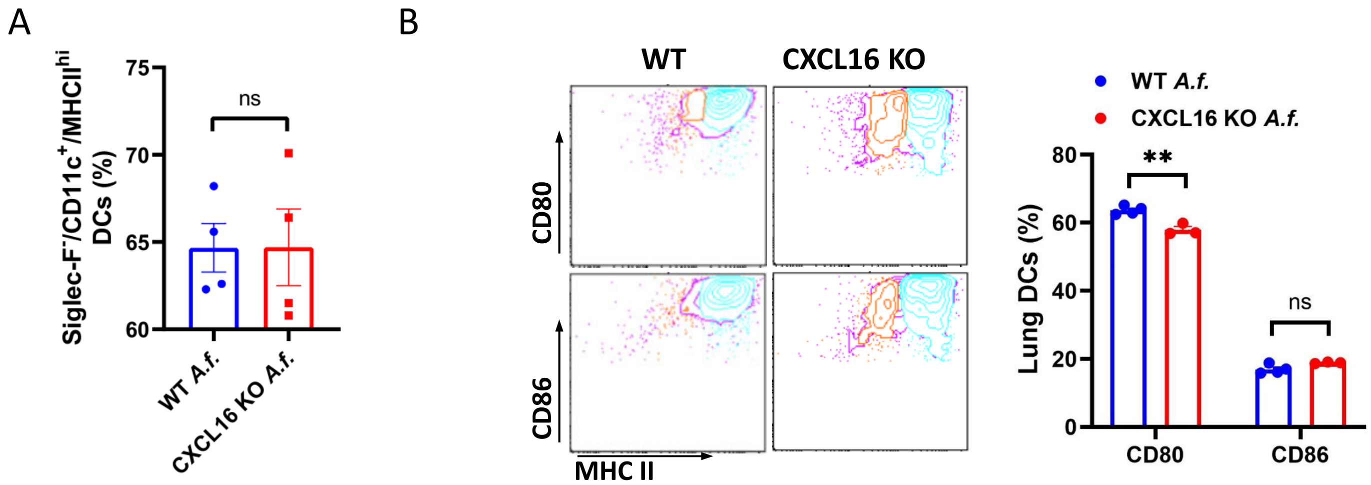


Supplemental figure 5. The phenotype of DCs by flow cytometry. (A) Percentage of CD11c+/Siglec-F−/MHC IIhi total dendritic cells of the lung (n = 4). (B) Cell surface expression of CD80 and CD86 by lung CD11c+/MHC IIhi/Siglec-F− DCs from Aspergillus-challenged wild-type and CXCL16-knockout mice (n = 3). *P < 0.05, **P < 0.01 Aspergillus-challenged CXCL16-knockout mice versus wild-type mice. *A.f.* is stand for *Aspergillus*.

Supplemental figure 6

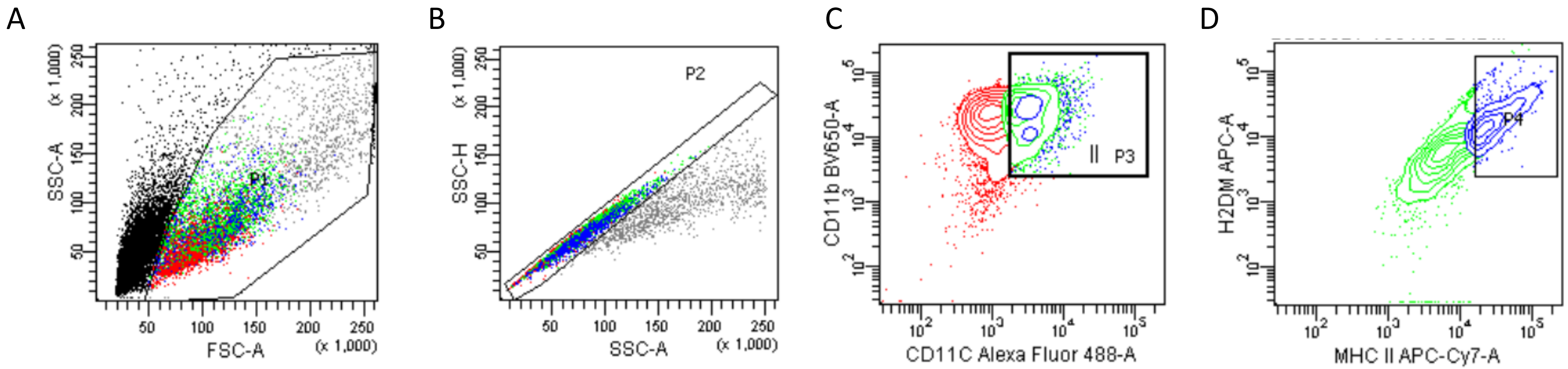


Supplemental figure 6. Gate for H2-DM. Gate for H2-DM by flow cytometry. (A) CXCL16 KO BMDCs or WT BMDCs induced by *Aspergillus* for 24h.(B) P2 for deadhesion cells of P1. (C) P3 for $CD11c^{+}CD11b^{+}$ cells of P2. (D) P4 for $H2\text{-}DM^{+}$ for MHC $^{hi}$ cells of P3.
