# Supplementary material for: CXCL16 knockout inhibit asthma airway inflammation by suppressing H2-DM molecular mediated antigen presentation
Source: Cell Death Discov. 2025 Mar 6;11:90. doi: 10.1038/s41420-025-02371-6 (PMC11885808; doi:10.1038/s41420-025-02371-6)
Supplement: Supplementary file 2 — Supplemental table 1 [file 41420_2025_2371_MOESM2_ESM.docx]

**SUPPLEMENTARY INFORMATION**

Supplemental table 1. Primers used for real-time PCR

| Gene | Forward Primer | Reverse Primer |
| --- | --- | --- |
| IL-13 | 5’-CAGCATGGTATGGAGTGTGG-3’ | 5’-GGAATCCAGGGCTACACAGA-3’ |
| Muc5AC | 5’-GTGCAGGGCTCAGTTCTTTC-3’ | 5’-TGGTCTCTGTTTTCGTGCTG-3’ |
| Muc1 | 5’-TACCAAGCGTAGCCCCTATG-3’ | 5’-ATTACCTGCCGAAACCTCCT-3’ |
| Gob-5 | 5’-AAACCAACGATGCAATCTCC-3’ | 5’-GTCATGGGAGTGGTTTGCTT-3’ |
| GAPDH | 5’-CATGGCCTTCCGTGTTCCTA-3’ | 5’-GCGGCACGTCAGATCCA-3’ |
| CD209a | 5’-AGTCCCAAAACCCTGCCAAA-3’ | 5’-CACCCAGGGGTAGAGATGGA-3’ |
| Clec12a | 5’- GCTTGTCCCTAGCTGCTCTC -3’ | 5’- TCGTGGCAGTCAGCAAATGA -3’ |
| Clec10a | 5’- AGGGCTGACAGCTTCGAAAA -3’ | 5’- CCTTCTGGCTCAAGTCTCGG -3’ |
| CD209g | 5’- TCAGTAGAGTCACACCCGCT -3’ | 5’- CCACCCATGCTAGGCAAACT -3’ |
| Clec7a | 5’- GGGTTTAGGAATCCTGTGCT -3’ | 5’- TACGGTGAGACGATGTTTGG -3’ |
| Clec4b1 | 5’- TACTCCTCAGCACCTGTTTCA -3’ | 5’- TCCTGCTCCTCCTGGCTAT -3’ |
| H2-DMb1 | 5’- ACCCCACAGGACTTCACATAC -3’ | 5’- GGATACAGCACCCCAAATTCA -3’ |
| H2-DMb2 | 5’- TGGGGTGCTGTCTAGATTGG -3’ | 5’- TGCAAGCGATGAATAAGGCT -3’ |
| Clec4e | 5’- AACCACATCCCACACTCCAC -3’ | 5’- TGGATGATTCCCCACCTGTC -3’ |
| TLR9 | 5’- CTCCAACCGTATCCACCACC -3’ | 5’- GAGAAGTGCAGGGGGCTAAG -3’ |
| TLR2 | 5’- TCTCTGCGACCTAGAAGTGGA -3’ | 5’- AGCAGAGAAGTGAAGCCCCT -3’ |
| NOD1 | 5’- TCGTCCTGCATCACTTCCAC -3’ | 5’- CCACATACCTGGCTCCGATA -3’ |
| NLRC3 | 5’- GGTTCCTTGCTTCATCGCAC -3’ | 5’- TGGTTTCATGGGAGTACCGC -3’ |
| Clec9a | 5’- CCAACAGGACACAGCATTGG -3’ | 5’-TGCAGTCACTACCTGAATGGAGA-3’ |
| TLR4 | 5’- GTGCCAGTCAGGGTCATTCA -3’ | 5’- GTTGAAAACTCCCCAGCCCT -3’ |
| Clec4n | 5’- ATTATGGACCAGCCCAGTAG -3’ | 5’- CGAAAGACCCAGGAAGTAAG -3’ |
| Olr1 | 5’- CAAGATGAAGCCTGCGAATGA -3’ | 5’- ACCTGGCGTAATTGTGTCCAC -3’ |
| ly86 | 5’- GGACCAGCCCAAGTTCTCA -3’ | 5’- CCACAGTAGCACGGTTTTCAT -3’ |
| Clec6a | 5’- TGGACCAGCCCAGTAGAAG -3’ | 5’- GACCCAGGAAGTAAGAAAGTGA-3’ |

| Muc5AC, indicates Mucin-5AC; Gob-5/ Clca3, Chloride channel, calcium activated, family member 3, GAPDH, glyceraldehyde 3-phosphate dehydrogenase; TLR4, Toll like receptor 4; |
| --- |
